# Brain health PRO/santé cerveau PRO: The development of a web-based program for dementia literacy and risk factor reduction

**DOI:** 10.1016/j.tjpad.2025.100206

**Published:** 2025-05-19

**Authors:** Alex Bahar-Fuchs

**Affiliations:** SEED Lifespan, School of Psychology, Deakin University, Melbourne, Australia

Neurocognitive disorders associated with neurodegenerative and vascular diseases represent one of the most pressing global health challenges, exerting immense emotional, societal, and economic burdens. Currently, around 55 million people globally live with dementia, and without substantial breakthroughs, this number is projected to soar to approximately 139 million by 2050, disproportionately affecting low- and middle-income countries (WHO, [5]).

Encouragingly, after decades dominated by a fatalistic view of cognitive decline as an unavoidable aspect of aging and with a palliative approach as the primary framework for the care of people with dementia, the past 15 years have heralded transformative advances in dementia research and care. Driven by robust epidemiological evidence demonstrating that lifestyle factors play a major role in dementia risk, prevention-focused research has rapidly proliferated, highlighting lifestyle changes as a key to reducing dementia risk [3]. Indeed, international initiatives such as the FINGER study and its global expansion through the World-Wide FINGERS network underscore the effectiveness and relevance of multidomain lifestyle interventions in dementia risk reduction [2].

Central to all prevention efforts, including dementia prevention, is the notion of empowering individuals to develop greater agency in managing their own health, including their cognitive health, through increased awareness and literacy. Digital platforms have emerged as scalable and customizable tools, adapting interventions to diverse groups' specific needs. Crucially, the effectiveness of such platforms is significantly enhanced when guided by robust theoretical foundations and transparently reported methodological rigor. This represents an important evolution in dementia prevention approaches, particularly when considering the scale and diversity of populations at risk globally.

In this issue of the Journal of Prevention of Alzheimer’s Disease, the study by Sylvie Belleville and colleagues exemplifies precisely this approach. Their manuscript represents an innovative and timely contribution, systematically addressing several critical gaps in the dementia prevention field. Although the importance of lifestyle and non-pharmacological treatments (NPTs) in dementia prevention is widely recognized, NPTs are not always designed and/or delivered against a sound theoretical frameworks and rigorous methodologies, issues which contribute to substantial heterogeneity in research findings, difficulty synthesizing the evidence, and to a reproducibility crisis [4]. Belleville et al.’s work stands out with commendable adherence to scientific rigor through meticulous application of the Intervention Mapping Approach (IMA), a dominant, systematic framework designed to integrate theory explicitly into health intervention development while also espousing the principle of co-design.

I was particularly impressed by the team's careful implementation of IMA guidelines, which facilitated explicit articulation of logic models clearly linking personal and environmental determinants, targeted behavioral risk factors, cognitive health outcomes, and quality-of-life considerations. This methodological clarity is exceptional and significantly enhances intervention transparency and replicability. Furthermore, Belleville and colleagues integrated key personalization principles, such as tailored risk profiling and individualized goal setting, thus reinforcing user engagement and relevance. Their detailed logic models not only offer clarity on the underlying intervention mechanisms but also provide a highly valuable template for future dementia prevention interventions.

This rigorous, theory-driven, online, multidomain intervention notably extends foundational earlier efforts, such as the Body Brain Life (BBL) program developed more than a decade ago [1]. Like Brain Health PRO, BBL employed theory-driven strategies, explicitly connecting behavioral determinants and interventions tailored to individual needs, demonstrating measurable dementia risk reduction among middle-aged participants. However, Brain Health PRO significantly advances this prior work by targeting older adults, expanding risk factor coverage (e.g., sensory impairment, sleep), and emphasizing user-centric co-design principles. Indeed, Belleville et al. involved end-users throughout the intervention development via the Citizen Advisory Group, ensuring enhanced relevance, acceptability, and engagement. This participatory design strategy demonstrates substantial progress in ensuring interventions are not merely theoretically robust but genuinely user-focused and practical.

Preliminary evaluation results indicating high acceptability were unsurprising, given the extensive design and engagement strategies employed. However, findings revealed intriguing behavioral patterns among participants, notably the prioritization of cognitive engagement, even in the absence of risk in this domain for most participants. This raises important considerations regarding balancing user autonomy and targeted intervention effectiveness, ensuring fidelity to theoretical assumptions underpinning multidomain interventions. Additionally, the gamified incentive system intended to bolster motivation did not yield anticipated improvements in engagement, underscoring complexities inherent in translating stakeholder insights into effective behavioral strategies and the challenges in predicting participant behaviors and preferences.

While Belleville et al.’s work is undoubtedly exemplary, some limitations merit discussion. Dementia risk reduction studies often recruit homogeneous, highly educated samples, and this feasibility study similarly reflects such demographic constraints. Future work should seek broader participant diversity to enhance generalizability. Moreover, despite incorporating personalization, the project may have overlooked opportunities to leverage recent revolutionary advances in artificial intelligence (AI), particularly machine learning algorithms and conversational AI agents. Such technological advancements offer groundbreaking potential for fully individualized interventions, substantially enhancing scalability and precision simultaneously. Leveraging AI to dynamically assess individual risk determinants, specific barriers to behavior change, and corresponding tailored intervention strategies could represent a pivotal evolution in dementia prevention efforts, addressing individualized needs at a scale previously unimaginable.

Looking ahead, a compelling question emerges regarding multidomain interventions' future relevance as precision medicine and individualized risk assessment evolve. Currently, multidomain approaches are comprehensive strategies to broadly mitigate dementia risk due to our limited ability to precisely identify individual risk contributions. However, as precision medicine advances, leveraging sophisticated predictive analytics, it remains an open question whether targeted, individualized interventions may eventually supplant traditional multidomain approaches, making the latter less central. Nonetheless, multidomain approaches currently remain essential precisely because they represent a pragmatic response to incomplete knowledge about individual risk pathways.

In conclusion, Belleville and colleagues have delivered a commendable, rigorous, and theoretically grounded intervention that represents a new benchmark in dementia prevention research. Their ambitious and meticulously executed work not only enriches the scientific evidence base but also provides a replicable model for future interventions. Fellow researchers should be inspired by this exemplary demonstration of scientific rigor, stakeholder collaboration, and methodological transparency, leveraging these principles as foundational pillars for future innovation and advancement in dementia risk reduction.

## Generative AI declaration

During the preparation of this editorial, the author used a ChatGPT in order to improve expression and readability. After using this tool, the author carefully reviewed and edited the content as needed and takes full responsibility for the content of the publication.

## Conflict of interests declaration

Alex Bahar-Fuchs is an employee of both Deakin University and NewDays and provided consulting services to CogniFit.

## CRediT authorship contribution statement

**Alex Bahar-Fuchs:** Writing – original draft, Writing – review & editing.

## Declaration of competing interest

The authors declare the following financial interests/personal relationships which may be considered as potential competing interests: Alex Bahar-Fuchs reports a relationship with NewDays that includes: consulting or advisory and employment. If there are other authors, they declare that they have no known competing financial interests or personal relationships that could have appeared to influence the work reported in this paper.
